# Engineering *Escherichia coli* FAB system using synthetic plant genes for the production of long chain fatty acids

**DOI:** 10.1186/s12934-019-1217-7

**Published:** 2019-10-03

**Authors:** Elias Kassab, Monika Fuchs, Martina Haack, Norbert Mehlmer, Thomas B. Brueck

**Affiliations:** Werner Siemens-Chair of Synthetic Biotechnology, Department of Chemistry, Technical University of Munich, 85748 Garching, Germany

**Keywords:** Type-II fatty acid synthase, Fatty acid biosynthesis, Long chain fatty acids, Heterologous expression, Chloroplast, β-Ketoacyl-[acyl carrier protein] synthase I and II, *Escherichia coli*, *Arabidopsis thaliana*

## Abstract

**Background:**

Sustainable production of microbial fatty acids derivatives has the potential to replace petroleum based equivalents in the chemical, cosmetic and pharmaceutical industry. Most fatty acid sources for production oleochemicals are currently plant derived. However, utilization of these crops are associated with land use change and food competition. Microbial oils could be an alternative source of fatty acids, which circumvents the issue with agricultural competition.

**Results:**

In this study, we generated a chimeric microbial production system that features aspects of both prokaryotic and eukaryotic fatty acid biosynthetic pathways targeted towards the generation of long chain fatty acids. We redirected the type-II fatty acid biosynthetic pathway of *Escherichia coli* BL21 (DE3) strain by incorporating two homologues of the beta-ketoacyl-[acyl carrier protein] synthase I and II from the chloroplastic fatty acid biosynthetic pathway of *Arabidopsis thaliana*. The microbial clones harboring the heterologous pathway yielded 292 mg/g and 220 mg/g DCW for KAS I and KAS II harboring plasmids respectively. Surprisingly, beta-ketoacyl synthases KASI/II isolated from *A. thaliana* showed compatibility with the FAB pathway in *E. coli*.

**Conclusion:**

The efficiency of the heterologous plant enzymes supersedes the overexpression of the native enzyme in the *E. coli* production system, which leads to cell death in *fabF* overexpression and *fabB* deletion mutants. The utilization of our plasmid based system would allow generation of plant like fatty acids in *E. coli* and their subsequent chemical or enzymatic conversion to high end oleochemical products.

## Background

To circumvent the ecological impact of plant oil production for generation of oleochemical building blocks, there is an increasing industrial demand for microbial generated fatty acids derived from bacteria, yeast or algae [1, 2] The chain length and degree of saturation of renewable fatty acids channels their use into either the biofuel, pharmaceutical or chemical industry. Wilde type *E. coli* produces a small range of saturated and unsaturated fatty acids with a chain length ranging from C12 to C18. Compared to other organisms it is neither a lipid accumulating organism nor a direct producer of high value fatty acid derivatives. However, due to its ease of genetic manipulation, *E. coli* has been successfully modified for the production for high value lipids and has been established as a model for generation of tailor made fatty acids [1, 3–7].

The sustainable production of microbial fatty acids and their derivatives as a renewable alternative to petroleum and other natural hydrocarbons has been the research focus over the past decades [1, 3, 5–10]. *Escherichia coli*’s fatty acid biosynthesis pathway has been thoroughly investigated [3, 11, 12]. A profound understanding of the *E. coli* fatty acid biosynthesis pathway and the enzymes involved is the basis for the remodeling its biosynthetic capacity to generate tailor made fatty acid profiles [1, 13–17].

**Fig. 1:**
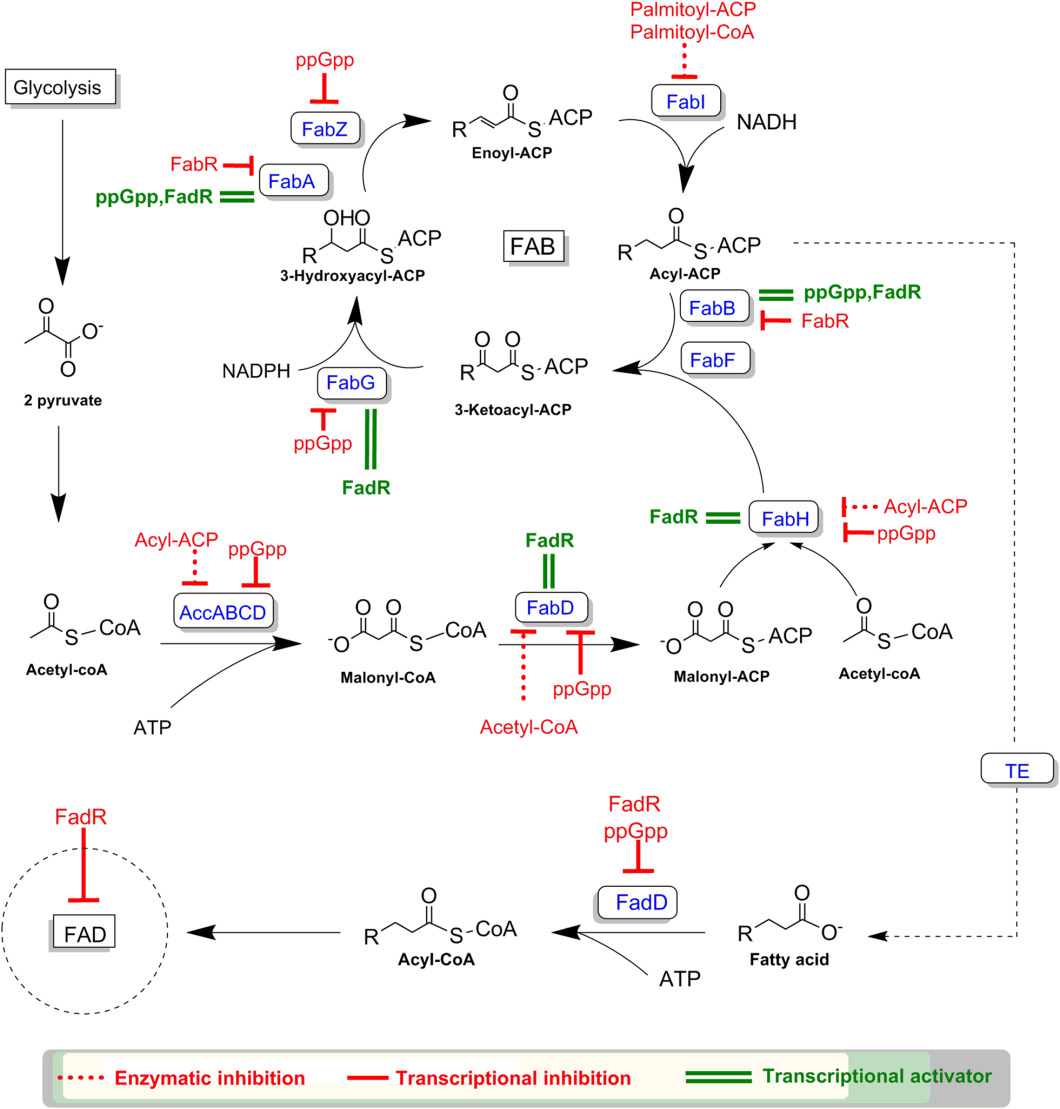
Model of de novo fatty acid biosynthesis and fatty acid degradation process in *Escherichia coli*

Prokaryotes like *E. coli* and prokaryote derived plant plastids both harbor the type-II fatty acid synthase (FAS), which is responsible for de-novo fatty acids biosynthesis. In *E. coli*, the FAS enzyme complex is made up of the FAB cluster, comprising the enzymes *fabA*, *fabB*, *fabD*, *fabF*, *fabG*, *fabH*, *fabI* and *fabZ*. The complete FAB complex is responsible for fatty acid production from Acetyl-CoA and the subsequent elongation using Malonyl-ACP (Acyl carrier protein) (Fig. 1). The natural fatty acid distribution ranges from C12 to C18 saturated and unsaturated fatty acids, with low amounts of vaccinate (C18:1). The formation of 3-Ketoacyl-ACP is catalyzed by the 3-Ketoacyl-ACP synthases (FabH, FabB and FabF) through the condensation of an acyl-ACP with malonyl-ACP. The first condensation reaction of acetyl-CoA and Malonyl-ACP is performed by FabH, initiating the first cycle of elongation. The proceeding two carbon elongation steps are performed solely by FabB and FabF (Fig. 1) [1, 14, 18, 19]. Unlike plants, wild type *E. coli* does not possess a native desaturase [12], therefore the synthesis of unsaturated fatty acids is catalyzed anaerobically by FabA and FabB [1, 12]. FabA is specific towards the 10-carbon acyl chain intermediate (3-hydroxydecanoyl-ACP) and introduces a double bond at the 10th carbon yielding trans-2-decenoyl-ACP which is then further isomerized by FabA to cis-3-decenoyl-ACP. FabB subsequently catalyzes the elongation of cis-3-decenoyl-ACP, which is the rate limiting step in unsaturated fatty acid synthesis. The overexpression of either FabA or FabB alone does not improve unsaturated fatty acid concentrations [1, 12].

Both FabB and FabF show activity towards saturated fatty acyl-ACPs up to C14, however FabB has low activity towards C14:0, while FabF only shows low activity towards C16:0 [1, 12, 19]. Regarding the synthesis of unsaturated fatty acids, FabB performs the elongation steps up to C16:1, while FabF only catalyzes the last elongation step leading to cis-vaccenic acid (C18:1). Due to the temperature sensitive nature of the FabF protein, which is expressed and active at low temperatures [20], FabB is more active and abundant [1, 12, 19]. The overexpression of FabB alone does not improve fatty acid titers and its deletion causes auxotrophy for unsaturated fatty acids. The overexpression of FabF is lethal to *E. coli* probably due to the high level of the enzyme both binding and inhibiting the function of FabD thus blocking the FAB cycle and increasing malonyl-CoA levels. The deletion of *fabF* inhibits C18:1 and C18:0 fatty acid production [1, 12, 19, 21, 22].

The fatty acid biosynthesis process in *E. coli* has been extensively investigated and modified in order to increase the total fatty acid titers, shifting the fatty acid production towards a specific chain length or altering the degree of saturation of the cellular fatty acid pool. Such metabolically engineered *E. coli* strains have made use of different ACP-Thioesterases, for example, the leaderless version of the native *E. coli* TesA (the leaderless version possesses a broader substrate preference) or the *Arabidopsis thaliana* AtFatA [10, 23]. Most thioesterases have a wide range of activity towards ACP-fatty acid chain length with a preferred specificity towards a specific chain length. Thioesterases provide a metabolic sink by deregulating the negative feedback mechanism of ACP bound fatty acids rather than specifically altering the FAB pathway [24]. Expression levels of each thioesterase must be carefully optimized since a slight increase above the optimal concentration leads to the inhibition of FFA production [23, 25]. Other engineered strains have deleted genes of the FAD pathway in order to prevent fatty acid degradation. Specifically, the deletion of *fadD* (fatty-acyl-CoA ligase) or *fadE* the first enzyme in the fatty acid oxidation cycle inhibit fatty acid catabolism, thereby leading to intracellular fatty acid accumulation. However, the fragile balance of the FAB pathway and its strict regulation have left little or no option for manipulation.

*KAS*I and *KASII* (β-ketoacyl-[acyl carrier protein] synthase I and II) are the respective homologues of *fabB* and *fabF* in plant chloroplasts [26–28]. Both *A. thaliana* KAS enzymes share a mere 36% identity to their *E. coli* homologues, with the active site and catalytic triad being conserved in both species. Furthermore, KASII is not temperature sensitive as its counterpart, FabF, in *E. coli* [26].

*Escherichia coli* has an enormous in vitro potential for fatty acid synthesis, however several bottlenecks limit its in vivo production capacity [29, 30]. In this study, we address one of the bottlenecks and report the successful manipulation of the very heavily modulated FAB elongation system in *E. coli* driving the already high flux pathway towards the production of longer chain fatty acid. We successfully implemented the ability of chloroplastic *A. thaliana* enzymes to complement and synergistically function with the *E. coli* FAB system in the selective production of long chain fatty acids.

## Results

### Expression of KASI and KASII in wildtype BL21DE3

Expression of both plant enzymes in wild type *E. coli* BL21 (DE3) resulted in an increase in the intracellular fatty acid pool. Moreover, we observed a shift in the fatty acid distribution relative to the wild type BL21 (DE3) strain harboring an empty pet28a-plasmid.

In the KASII clones, we detected an increase in unsaturated fatty acids, particularly long chain fatty acids (i.e. C16:1 and C18:1) with regard to the wildtype BL21 (DE3) strain.

**Fig. 2:**
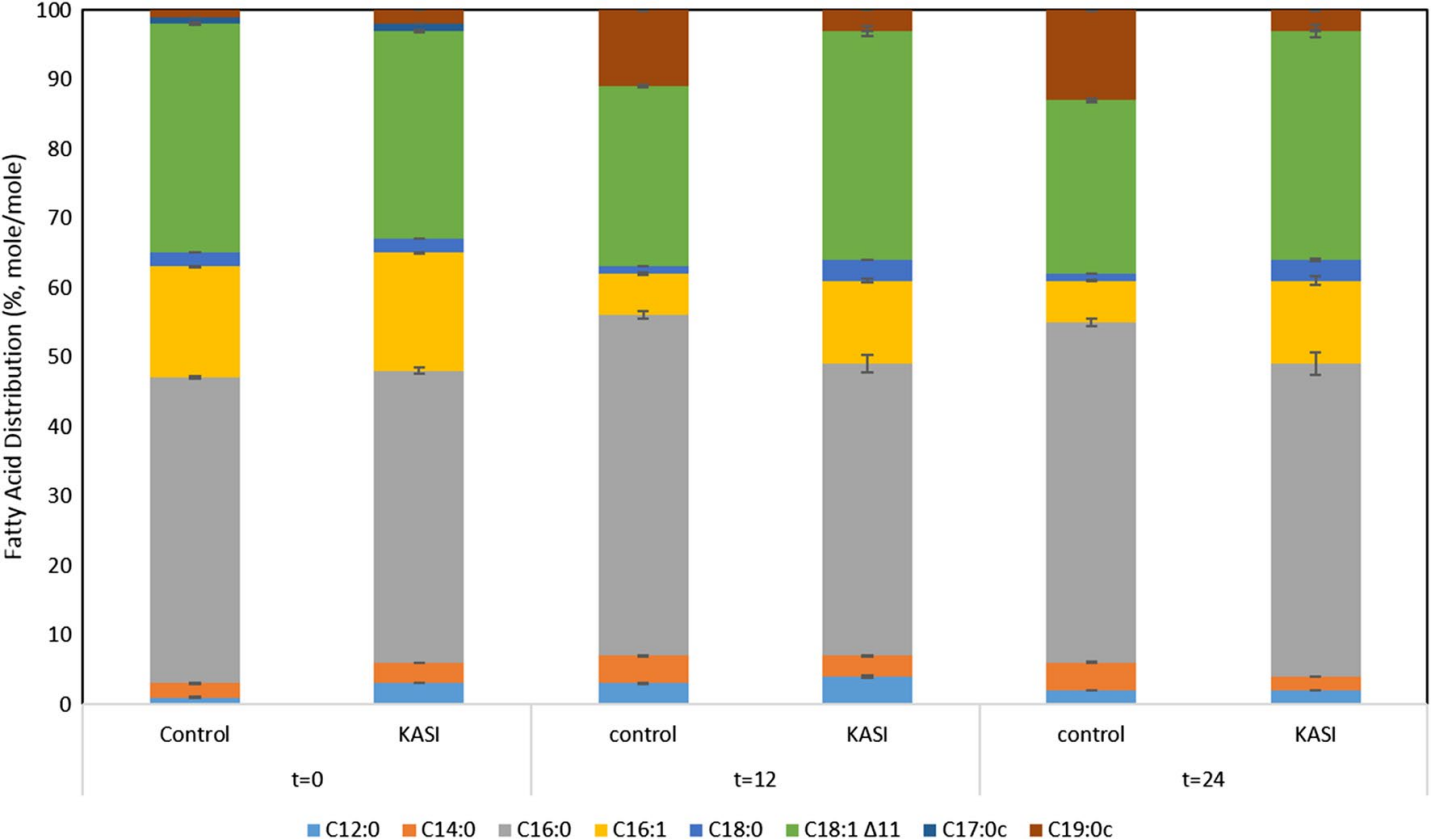
Fatty acid distribution in mole percent (% mole/mole) of “control”: wild type *Escherichia coli* BL21 (DE3) versus “KASI”: BL21 (DE3) expressing pET28aKASI at different time intervals. T = 0: directly before induction with IPTG, t = 12: 12 h after induction with IPTG, t = 24: 24 h after induction with IPTG. All fatty acid values are the average of at least three biological replicates with the associated standard deviation indicated

By comparison, KASI clones have shown improved fatty acids concentrations with specific increases in C14:0, C16:0, C16:1, C18:0 and C18:1 fatty acids. Saturated fatty acids constituted 52% (mol/mol) of the total fatty acid pool in our KASI clone versus 56% (mol/mol) in the control (Fig. 2). Amounts of C18:1 and its cyclic propane derivative C19:0c constitute 36% (mol/mol) of total fatty acids in our KASI clone versus 38% (mol/mol) in the control. However, we have noticed a decrease in cyclopropane conversion in our KASI strain, from 13% (mol/mol) in the control down to 3% (mol/mol) in our clone. Moreover, palmitoleic acid constitutes up to 12% (mol/mol) of the total fatty acid pool in our KASI clone versus 6% (mol/mol) in the control. Notably, unsaturated fatty acids constitute 44% (mol/mol) of total fatty acids in the wild type strain versus 48% (mol/mol) in the KASI clone.

### KASI and KASII in *fadD* knockouts

**Fig. 3:**
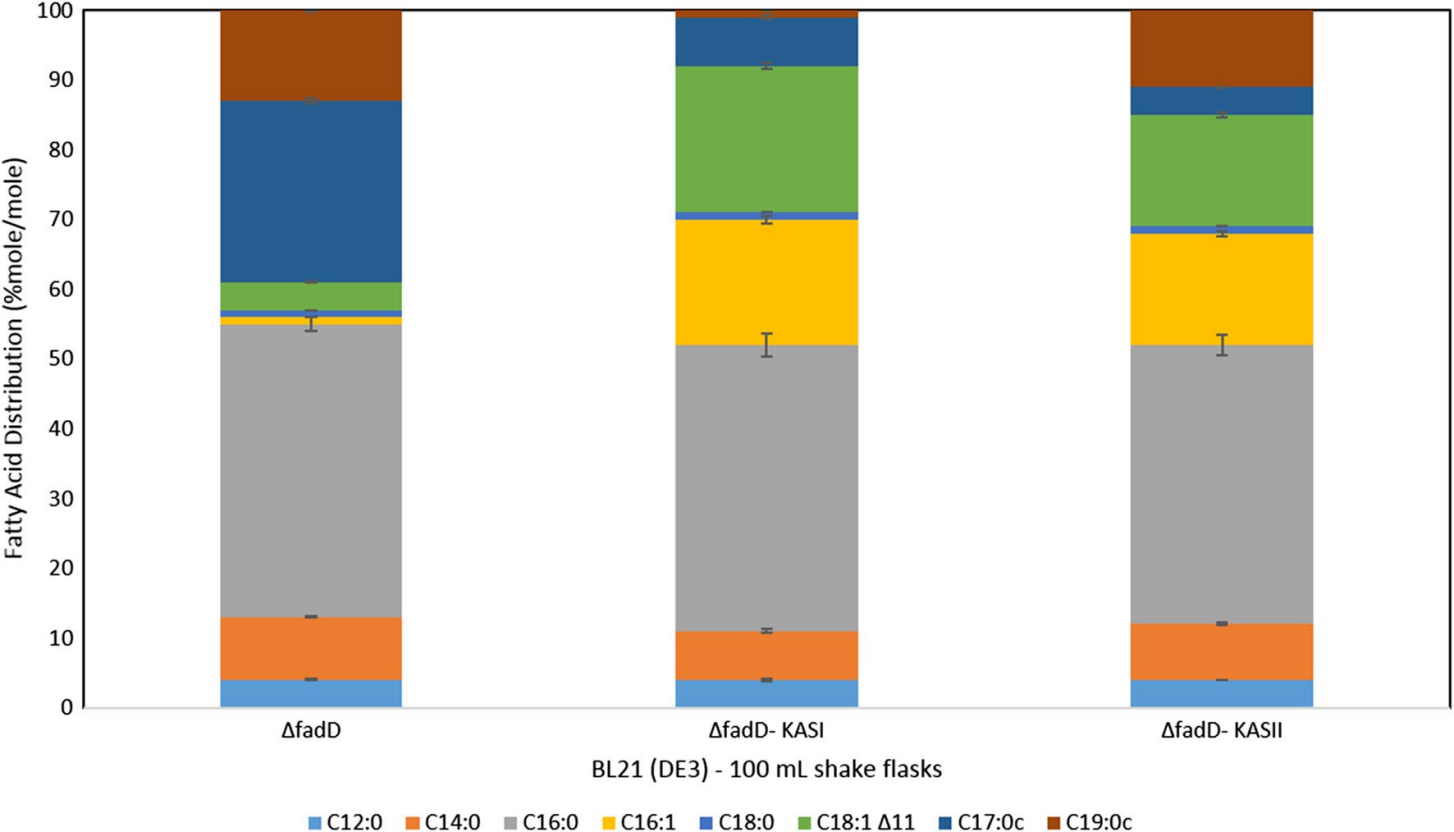
Fatty acid distribution in mole percent (% mole/mole) of *E. coli* BL21 (DE3) *ΔfadD* pET28a− versus BL21 (DE3) *ΔfadD* expressing pET28aKASI and pET28aKASII separately. All fatty acid values are the average of at least three biological replicates with the associated standard deviation indicated

In the ΔfadD strain a different fatty acid distribution was observed with a slight increase in fatty acid concentrations relative to the control strain, which is in accordance with previous literature [31, 32]. Our ΔfadD KASI clone showed a significant increase in overall fatty acid accumulation (see Additional file 1: Table S3). Interestingly, cis-vaccenic acid did comprise 4% (mol/mol) of the total fatty acids in the ΔfadD strain compared to 21% (mol/mol) in our ΔfadD KASI clone. The total amount of generated cis-vaccenic acid including the proportion converted to C19:0c, in the ΔfadD strain amount to 17% (mol/mol) of the total fatty acids produced versus 24% (mol/mol) in our ΔfadD KASI clone (Fig. 3). In our control strain, we observed an almost complete conversion of palmitoleic acid (1% mol/mol) to its cyclopropane fatty acid form (26% mol/mol). The ΔfadD KASI clone retains 18% (mol/mol) of the total fatty acids in the palmitoleic acid form versus 7% in the cyclopropane fatty acid form.

The ΔfadD KASII clone retains 16% of the total fatty acids in palmitoleic form versus 4% in cyclopropane form. Moreover, the fatty acid distribution in the ΔfadD KASII clone shows 30% of its total fatty acids are C18 versus 23% in the ΔfadD KASI clone and 18% in the ΔfadD strain. We observe an increase in the total amounts of unsaturated fatty acids from 44% in the ΔfadD strain, to 47% in the ΔfadD KASI strain to 49% in the ΔfadD KASII strain (Fig. 3).

### KASII in *fabF* knockouts

**Fig. 4:**
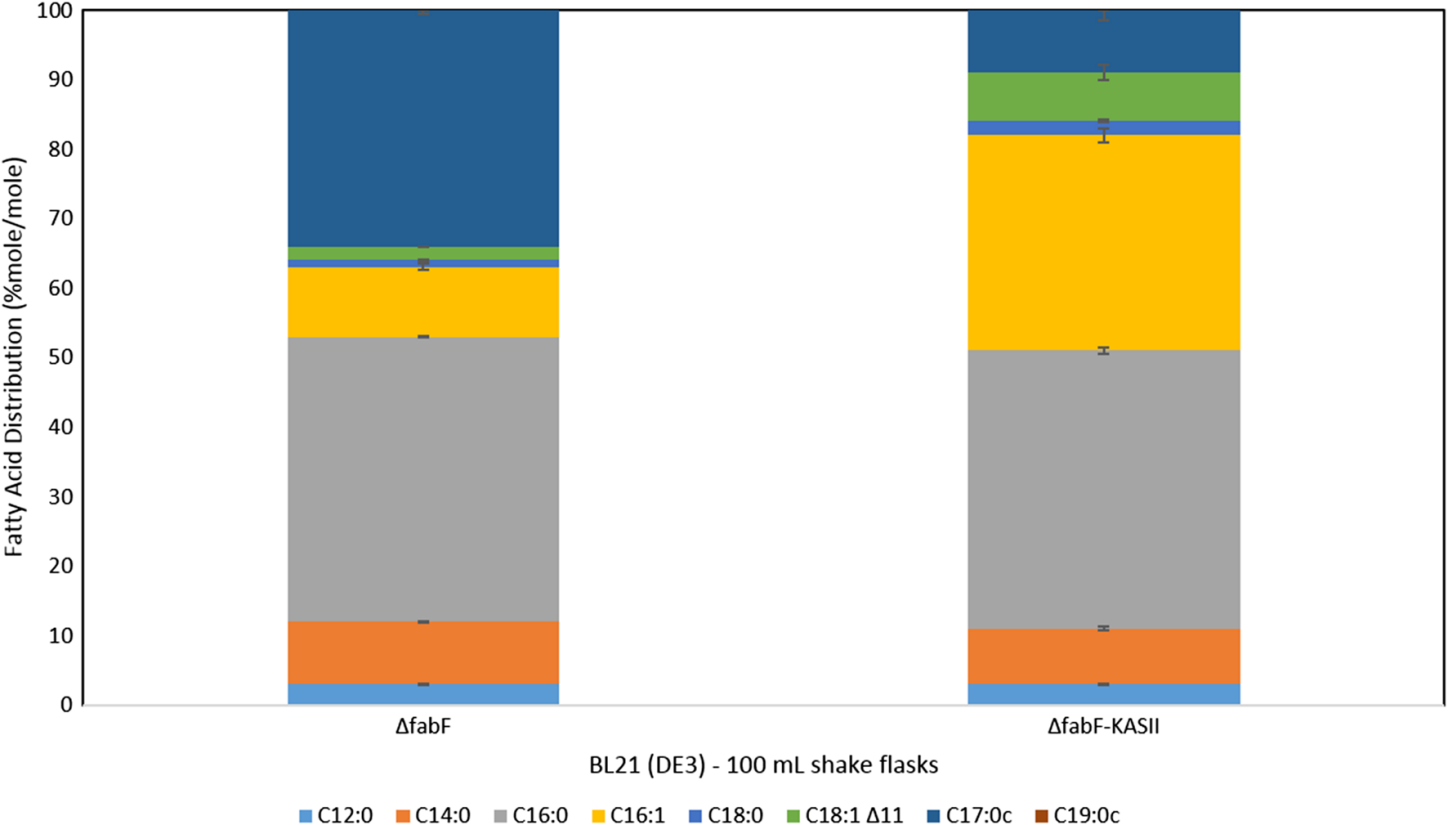
Fatty acid distribution in mole percent (% mole/mole) of *E. coli* BL21 (DE3) *ΔfabF* pET28a− versus BL21 (DE3) *ΔfabF* pET28aKASII. All fatty acid values are the average of at least three biological replicates with the associated standard deviation indicated

The generated *Δ*fabF strain, due to the loss of the FabF protein responsible for the elongation of 16 carbon atom acyl ACPs, showed a significant decrease in C18 fatty acid species (see Additional file 1: Table S4), which is in accordance with previous literature [22]. The deletion also resulted in a shift in fatty acid distribution where palmitic and palmitoleic fatty acids accumulated making up more than 70% of the total fatty acids. After the cloning of the *Arabidopsis thaliana* beta ketoacyl acp synthase II (KASII) gene, a significant increase of C18 fatty acid levels were observed, 3.5-fold increase compared to the *Δ*fabF strain, in addition to a 2.6-fold increase in palmitoleic acid. The fatty acid distribution of our *Δ*fabF KASII clone highly varies from that of the *Δ*fabF strain as seen in Fig. 4. It is noteworthy to state that C19:0c fatty acids were nonexistent in both strains, and a significant reduction in C17:0c fatty acids, from 34% in the ΔfabF strain to 9% in the ΔfabF KASII clone, has been observed. No significant change in fatty acid concentration could be detected.

### KASI in *fabB* knockouts

According to literature, the deletion of *fabB* causes auxotrophy to saturated fatty acids and inhibits growth [20]. We attempted to knock out *fabB* in our BL21 (DE3) strain using the same procedure as the *fabF* knock out. However, we were unable to retrieve viable colonies that had the gene knocked out. We assume that *fabB* is crucial for survival in BL21 (DE3) even in the presence of solid media supplemented with saturated fatty acids including oleic acid.

### KASI and KASII fermentation

The shake-flasks experiments described above clearly indicate that successful cloning of plant KAS enzymes and their incorporation within the FASII system is feasible in recombinant *E. coli*. To evaluate the performance of these enhanced strains at an industrial-process, fed-batch fermentations were performed in a bioreactor with defined media.

**Fig. 5:**
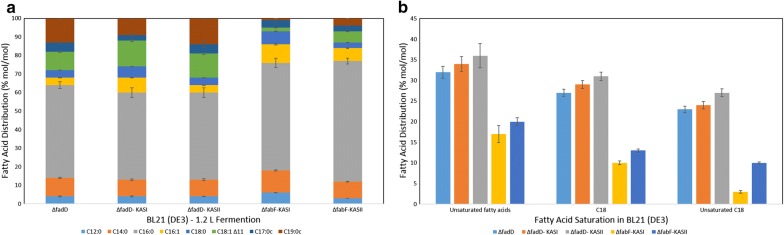
**a** Fatty acid distribution in mole percent (% mole/mole) of the 1.3 L fermentation of *E. coli* BL21 (DE3) *ΔfadD* expressing an empty pET28a vector, pET28aKASI and pET28aKASII respectively and of *E. coli* BL21 (DE3) *ΔfabF* expressing pET28aKASI and pET28aKASII respectively. **b** Comparison of total Unsaturated FA, total C18 FA and unsaturated C18 FA distribution (% mole/mole) of the same clones in part **a**. All fatty acid values are the average of at least three biological replicates with the associated standard deviation indicated

All clones were induced under nitrogen limiting conditions to enhance lipogenesis. Samples were collected after 48 h of induction with IPTG. Total fatty acid concentration of the KASI and KASII clone in ΔfadD strain reached 292 mg/g And 220 mg/g DCW (see Additional file 1: Table S5 and S6). In particular, ΔfadD KASI showed an 80% increase in fatty acid concentrations, while ΔfadD KASII also showed a 40% increase compared to ΔfadD strain. The total amount of unsaturated fatty acids was higher in the clones expressing the KASII protein compared to all other clones (Fig. 5b).

Our fermentation results demonstrate that the ΔfabF strain’s C18 fatty acids constitute only 4–5% of the total fatty acids. Moreover, the KASII complemented strain shows an increase of C18 fatty acid species to by about 13% with regard to the total fatty acid pool (Fig. 5a). By contrast, complementation with KASI resulted in 10% of total fatty acid species being C18 fatty acids. It is noteworthy that the increase of C18 fatty acids in the KASI strain was stearic acid, whereas the increase in the KASII strain was cis-vaccenic acid. This has been also observed in the ΔfadD strain containing KASI and KASII. Most notably, neither the increase in fatty acid concentrations nor the shift in the fatty acid profile was lethal to the cell. This is based on the fact that control and mutant strains have all exhibited almost the same growth rate with almost the same final OD_600_).

## Discussion

Decades of work have been dedicated to the biochemical optimization and metabolic engineering of *E. coli* aiming to increase fatty acids yield with tailored profiles [1, 3, 4, 33]. A common approach in early metabolic engineering studies involved addressing the bottlenecks observed in the native *E. coli* FAS system and its tight regulation, optimizing precursor supply, redirecting the carbon flux into a product of interest and downregulating or eliminating competing pathways [1, 3–6, 29, 34]. Despite this body of work, additional improvement in fatty acid production yield and profile specificity still requires further examination of pathway limitations and alleviation of the tight feedback regulations. However, these strategies are time-consuming and necessitate fine-tuning individual pathway components and targeting each rate-limiting factor until the desired goal is reached [3, 5, 6, 8, 11, 16].

In the fatty acid biosynthetic pathway of *E. coli*, both FabB and FabF proteins function in the condensation reaction and are the first enzymes in the fatty acid elongation system [1, 14]. Their expression level and specificity towards the substrate greatly affects the fatty acid profile of the organism. The FadD protein is responsible for the initial step of fatty acid degradation by adding a CoA group to a free fatty acid. In comparison, in plant chloroplasts, three isoforms of ketoacyl-ACP synthase exist, encoded by the *KASI*, *KASII* and *KASIII* genes [26, 27]. The KASIII enzyme is responsible for the initial condensation reaction and accepts only acetyl-CoA as the priming unit. In contract, KASI and KASII use beta-ketoacyl-[acp] as the priming unit and are crucial for further elongation of the carbon chain from C4 to C16 for KASI and from C16 to C18 for KASII [26, 27].

In the present study, wild type *E. coli* Bl21 (DE3) strain was genetically modified for the purpose of enhancing the strictly modulated FASII system for the production of long chain fatty acids. *KASI* and *KASII* genes were synthesized following codon optimization for optimal expression and the endogenous *E. coli* *fadD* was knocked out to inhibit the degradation of free fatty acids [1, 3, 4]. We demonstrate for the first time that eukaryotic, plant KAS enzymes are functional within the native FASII system of *E. coli*, allowing for a definitive shift in the fatty acid profile.

Previous studies showed that the overexpression of FabB alone or in combination with other enzymes of the FAS system in *E. coli* did not improve fatty acid concentrations [1, 12]. In this study, we demonstrate that the overexpression of the *A. thaliana KASI* gene, the homologue of FabB, in wild type *E. coli* shows an improvement in the overall production of saturated fatty acids with increases in myristic, palmitic acid, and stearic acid in addition to cis-vaccenic acid. We were unable to knock out the *fabB* gene at its locus. However, at present there is only one literature precedence that reports a *fabB* deletion mutant [35], which may be an indication of the difficulties to generate a knock-out at this particular locus. It is noteworthy that the lack of the *fabB* deletion mutant does not limit the conclusions drawn from our data.

Overexpressing the native *E. coli* FabF was previously reported to inhibit the FASII system and therefore fatty acid production. This lead to an intracellular accumulation of malonyl-CoA, which induced cell death [22]. FabF is reported to complex with FabD. Overexpression of FabF leads quantitative elimination of free FabD proteins in the cell, which stalls fatty acid synthesis. This hold true since FabD requires sequential complexation with FabH and FabB in order to activate the FAS system [22]. In turn, this drives malonyl-CoA accumulation, which in turn also inhibits fatty acid production and is lethal to the cell [22].

The overexpression of FabF has been widely applied to increase malonyl-CoA pools for subsequent production of polyketides and flavonoids [21]. Cloning of the *A. thaliana KASII* gene—the homologue of *fabF*—in *E. coli* was not lethal to the cell and did not inhibit the endogenous fatty acid synthase. Hence KAS II overexpression allowed fatty acid synthesis to commence without accumulation of manolyl CoA. Overexpression of KASII additionally provided an improvement in the overall fatty acid concentrations, particularly driving up the palmitoleic and cis-vaccenic acid pool. In contrast to its *E. coli* equivalent FabF [20], the *A. thaliana* KASII is active over the broad temperature range (25–37 °C) assayed in this study (data not shown).

In accordance with the literature, knocking out the *fabF* gene severely inhibited the production of C18 species [1]. The expression of the KASII protein in the *fabF* knock out strain not only complemented the loss of function of the endogenous protein, but it also leads to a significant increase in both palmitoleic and cis-vaccenic acids relative to the wild type *E. coli*. The expression of *KASI* did not complement the loss of the native *fabF* gene, however, it confirmed the pattern of *KASI* expression in other clones where significant increases in palmitoleic and stearic acid were measured.

The complementation of ΔfabF strain with KASII, suggests that chloroplast enzymes, while only sharing 33% identity with the native *E. coli* enzymes, function efficiently in the prokaryotic system. Moreover, our cumulative data suggests that the overexpression of both KASI and KASII did not induce a transcriptional inhibition by FabR.

Several studies have extensively targeted the upstream processes of the fatty acid synthase system [8]. Whether, to increase malonyl-CoA levels, the main precursor for the FAS, by targeting the *AccABC* and *AccD* genes or by overexpressing or cloning a recombinant KASIII enzyme—the enzyme responsible for the initial precursor condensation in plants [8, 25]. However, these systems were limited at the second condensation reaction coordinated by the FabB and FabF enzymes. The second condensation reaction is a very rapid step, where elongation is fast and reaches its limit according to what substrate each enzyme can accept. In that regard, FabB was limited at C14, while FabF is only limited to accept C16 fatty acid species as substrates. The use of *A. thaliana* KASI and KASII enzymes drove the elongation step beyond the acceptance of C16 fatty acids as FAS substrates. This KAS dependent feature results in a significant change in the fatty acid distribution. KAS enzyme overexpression alone is not sufficient to drastically increase fatty acid concentrations. Surprisingly; together our synergistic plant and bacterial fatty acid biosynthesis system can improve fatty acid concentrations and distributions by cumulatively enhancing both upstream and downstream fatty acid biosynthesis processes. Most notably, both beta-ketoacyl synthases KASI/II isolated from *A. thaliana* are completely compatible with the *E. coli* FAB pathway. It is imperative to state that both enzymes have shown to be more effective than the overexpression of the native enzyme.

## Conclusion

*Escherichia coli* has become the model organism to study microbial fatty acid biosynthesis. However, while *E. coli* itself is not a dedicated oleaginous organism, extensive genetic engineering allowed for generation of respectable product titers. However, increasing the total fatty acid concentration and shifting the native fatty acid profile towards longer chain fatty acids with industrial application in the food and oleochemical industry remains a scientific challenge. This study has examined the effects of the plant derived, chloroplastic β-ketoacyl synthases (KAS) on the fatty acid biosynthesis of *E. coli*. We could demonstrate that KAS I and KAS II can complement the native fatty acid biosynthesis in *E. coli* and shift product profiles towards longer chain (C16–C18) type fatty acids. Interestingly, we these plant enzymes exert equivalent effects in *E. coli* as observed in their native plant source. In that respect, KAS I increases relative fatty acid concentrations of the cell, particularly promoting formation of C16–C18 type fatty acids. By contrast, KASII did not lead to a significant increase in the total fatty acid concentration but lead to a targeted relative increase of the C18:1, cis-vaccenic acid. Since, plant derived triglycerides mainly feature C16–C18 fatty acids, the integration of the plant derived KASI and II can mimic this fatty acid profile in *E. coli*. Therefore, utilization of our plasmid based system would allow generation of plant like fatty acids in *E. coli* and their subsequent chemical or enzymatic conversion to high end oleochemical products. One such example could be the conversion of cis-vaccenic acid to 10-hydroxy stearic acid using a dedicated vaccinate hydratase enzyme [36]. We are continuously probing and expanding our portfolio of *E. coli* variants for tailored fatty acid biosynthesis. In that regard, we focus on further extending the fatty acid chain length towards very long chain fatty acids, such as Eicosanoic acid (C20:0), Eicosenoic acid (C20:1) and Erucic acid (C22:1), which are in demand and associated with high pricing regimes in the production of cosmetics, lubricants and polymer industry [37, 38].

## Methods

### Genes and plasmids

cDNA sequences of both beta-ketoacyl-[acyl carrier protein] synthase I and II (*KASI* Gene ID: AT5G46290.3 and *KASII* Gene ID: AT1G74960.2) were obtained from The Arabidopsis Information Resource (TAIR), on www.arabidopsis.org. The chloropalstic transit peptide of both were predicted using *SignalP 4.1 Server* and removed from the mRNA sequence. The mature sequences were codon optimized for expression in *E. coli* and chemically synthesized by Eurofins Scientific. The genes were synthesized to include a 5′ *Bam*HI and a 3′ *Not*I restriction site that were later used to be cloned into the MCS of an empty pET28a vector. The cloned genes were confirmed by sequencing (Eurofins Scientific). All primers were synthesized by Eurofins Scientific and all plasmids were obtained from Novagen/Merk Millipore.

### Bacterial strains and growth conditions

All bacterial strains used were obtained from Merk Millipore. *E. coli* DH5 alpha strain grown at 37 °C in Luria–Bertani medium was used for cloning and plasmid amplification. *E. coli* BL21 (DE3) strain was used for expression and fatty acid production. Minimal M9 media supplemented with 0.4% glucose and a pH = 6.9 was used for the shake flask studies. For shake flask studies, all clones were grown at 37 °C with the appropriate antibiotics (Kanamycin 50 μg/mL) and induced at an OD600 of 0.6 with 0.05 mM IPTG (isopropyl-β-d-thiogalactopyranoside).

### Fatty acid methylation and analysis

Samples taken from shake flask and fermentation studies were centrifuged and subsequently washed twice with ddH_2_O. The samples were then lyophilized and equal amounts of dry cell weight were taken for analysis. Methanol transesterification according to the protocol of Griffiths et al. was used to directly convert dry cell biomass to fatty acid methyl esters (FAMES) 0.1 μL of each sample was injected into gas chromatograph–flame ionization detector (GC–FID) for separation and quantification. GC–MS was performed with the Thermo Scientific™ TRACE™ Ultra Gas Chromatograph instrument coupled to a Thermo DSQ™ II mass spectrometer and the Triplus™ Autosampler injector. The analysis was carried out using a Stabilwax^®^ fused silica capillary column (30 m × 0.25 mm, with a film thickness of 0.25 μm). The analysis was performed using the following temperatures: initial column temperature 50 °C, programmed to increase at a rate of 4 °C/min up to a final temperature of 250 °C. The carrier gas used was hydrogen at a constant flow rate of 35 mL/min. FAMEs Marine Oil Standard was used as a standard reference, containing 20 components from C14:0 until C24:1. At least three biological replicates were used for fatty acid analysis. The statistical differences and percent change presented in this manuscript are only shown for differences that were statistically significant (P < 0.05).

### Fermentation

The DASGIP^®^ 1.3 L parallel reactor system (Eppendorf AG) was used to perform parallel fermentations. A modified M9 media consisting of 8 g L^−1^ NH4Cl, 13.3 g L^−1^ KH2PO4, 1.24 g L^−1^ MgSO_4_·7H_2_O, 0.42 g L^−1^ FeCl^3−^·6H_2_O, 40 g L^−1^ Glucose was used as batch media. Fermenters were inoculated with an overnight pre-culture with a starting OD600 of 0.1. The cultivation temperature was kept constant at 37 °C. Initial stirring velocity and airflow was set to 200 rpm and to 0.2 volumes of air per volume of medium per min (vvm), respectively. Dissolved oxygen was maintained at 30% by successive increases of the stirrer velocity, the oxygen concentration, and eventually the airflow. A pH value of 7.00 was controlled by the addition of 6 M aqueous NaOH. A pH value shift above 7.05 initiated a feed shot of 40 mL. The feed solution consisted of 500 g L^−1^ glucose, 20 g L^−1^ MgSO4·7H_2_O, 2 mg L^−1^ thiamine–HCl, 16 mL 100× trace elements solution (5 g L^−1^ EDTA; 0.83 g L^−1^ FeCl^3−^·6H_2_O; 84 mg L^−1^ ZnCl2, 13 mg L^−1^ CuCl^2−^·2H_2_O, 10 mg L^−1^ CoCl^2−^·2H_2_O, 10 mg L^−1^ H_3_BO_3_, and 1.6 mg L^−1^ MnCl^2−^·4H_2_O) (pH = 7.00). Samples were taken at different time points to determine the OD600. Once the clones reached limitation due to the depletion of the nitrogen source, they were induced with 0.05 mM IPTG. Samples for fatty acid analysis were collected at different intervals.

## Supplementary information

**Additional file 1: Table S1.** Fatty acid distribution in μg/g DCW of wild type *Escherichia coli* BL21 (DE3) at different time intervals. T = 0: directly before induction with IPTG, t = 12: 12 h after induction with IPTG, t = 24: 24 h after induction with IPTG. All fatty acid values are the average of at least three biological replicates with the associated standard deviation indicated. **Table S2.** Fatty acid distribution in μg/g DCW of *Escherichia coli* BL21 (DE3) expressing pET28aKASI at different time intervals. T = 0: directly before induction with IPTG, t = 12: 12 h after induction with IPTG, t = 24: 24 h after induction with IPTG. All fatty acid values are the average of at least three biological replicates with the associated standard deviation indicated. **Table S3.** Fatty acid distribution in μg/g DCW of *Escherichia coli* BL21 (DE3) ΔfadD pET28a− versus BL21 (DE3) ΔfadD expressing pET28aKASI and pET28aKASII separately. Samples were collected 48 h after induction with IPTG. All fatty acid values are the average of at least three biological replicates with the associated standard deviation indicated. **Table S4.** Fatty acid distribution in μg/g DCW of *Escherichia coli* BL21 (DE3) ΔfadF pET28a− versus BL21 (DE3) ΔfadD expressing pET28aKASII. Samples were collected 48 h after induction with IPTG. All fatty acid values are the average of at least three biological replicates with the associated standard deviation indicated. **Table S5.** Fatty acid distribution in μg/mg DCW of the 1.3 L fermentation of *Escherichia coli* BL21 (DE3) ΔfadD expressing an empty pET28a vector, pET28aKASI and pET28aKASII respectively. Samples were collected 48 h after induction with IPTG. All fatty acid values are the average of at least three biological replicates with the associated standard deviation indicated. **Table S6.** Fatty acid distribution in μg/mg DCW of the 1.3 L fermentation of *Escherichia coli* BL21 (DE3) ΔfabF expressing pET28aKASI and pET28aKASII respectively. Samples were collected 48 h after induction with IPTG. All fatty acid values are the average of at least three biological replicates with the associated standard deviation indicated. **Table S7.** Growth analysis of M9 minimal media shake flask studies of wild type *Escherichia coli* BL21 (DE3) expressing pET28a−, pET28aKASI and pET28aKASII respectively. Absorbance values (OD600) are the average of at least three biological replicates. **Figure S1.** Growth curve of M9 minimal media shake flask studies of wild type *Escherichia coli* BL21 (DE3) expressing pET28a−, pET28aKASI and pET28aKASII respectively. Absorbance values (OD600) are the average of at least three biological replicates with the associated standard deviation indicated.

## Data Availability

All data generated or analyzed during this study are included in this published article and its Additional file. Additional data required is available from the corresponding author on reasonable request.

## Abbreviations

FA: fatty acid
FAB: fatty acid biosynthesis
FAS: fatty acid synthase
DCW: dry cell weight
ACP: acyl carrier protein
CoA: coenzyme A
KASI: β-ketoacyl-[acyl carrier protein] synthase I
KASII: β-ketoacyl-[acyl carrier protein] synthase II
FabB: 3-oxoacyl-[acyl-carrier-protein] synthase 1
FabF: 3-oxoacyl-[acyl-carrier-protein] synthase 2
FadD: fatty acyl-CoA ligase
