# Supplementary material for: Engineering Escherichia coli FAB system using synthetic plant genes for the production of long chain fatty acids
Source: Microb Cell Fact. 2019 Oct 3;18:163. doi: 10.1186/s12934-019-1217-7 (PMC6777021; doi:10.1186/s12934-019-1217-7)
Supplement: Supplementary file 1 — Additional file 1: Table S1. Fatty acid distribution in μg/g DCW of wild type Escherichia coli BL21 (DE3) at different time intervals. T = 0: directly before induction with IPTG, t = 12: 12 h after induction with IPTG, t = 24: 24 h after induction with IPTG. All fatty acid values are the average of at least three biological replicates with the associated standard deviation indicated. Table S2. Fatty acid distribution in μg/g DCW of Escherichia coli BL21 (DE3) expressing pET28aKASI at different time intervals. T = 0: directly before induction with IPTG, t = 12: 12 h after induction with IPTG, t = 24: 24 h after induction with IPTG. All fatty acid values are the average of at least three biological replicates with the associated standard deviation indicated. Table S3. Fatty acid distribution in μg/g DCW of Escherichia coli BL21 (DE3) ΔfadD pET28a− versus BL21 (DE3) ΔfadD expressing pET28aKASI and pET28aKASII separately. Samples were collected 48 h after induction with IPTG. All fatty acid values are the average of at least three biological replicates with the associated standard deviation indicated. Table S4. Fatty acid distribution in μg/g DCW of Escherichia coli BL21 (DE3) ΔfadF pET28a− versus BL21 (DE3) ΔfadD expressing pET28aKASII. Samples were collected 48 h after induction with IPTG. All fatty acid values are the average of at least three biological replicates with the associated standard deviation indicated. Table S5. Fatty acid distribution in μg/mg DCW of the 1.3 L fermentation of Escherichia coli BL21 (DE3) ΔfadD expressing an empty pET28a vector, pET28aKASI and pET28aKASII respectively. Samples were collected 48 h after induction with IPTG. All fatty acid values are the average of at least three biological replicates with the associated standard deviation indicated. Table S6. Fatty acid distribution in μg/mg DCW of the 1.3 L fermentation of Escherichia coli BL21 (DE3) ΔfabF expressing pET28aKASI and pET28aKASII respectively. Samples were collected 48 h afte [file 12934_2019_1217_MOESM1_ESM.pdf]

Additional file

Engineering Escherichia coli FAB system using synthetic plant genes for the production of long chain fatty acids.

Elias Kassab[1], Monika Fuchs[1], Martina Haack[1], Norbert Mehlmer[1], Thomas B. Brueck[1]*.

Table S1: *Fatty acid distribution in µg/g DCW of wild type Escherichia coli BL21 (DE3) at different time intervals. T=0: directly before induction with IPTG, t=12: 12 hours after induction with IPTG, t=24: 24 hours after induction with IPTG. All fatty acid values are the average of at least three biological replicates with the associated standard deviation indicated.*

BL21 (DE3) pET28a-						
Fatty acid distribution (µg/g)	T=0		T=12		T=24	
C12:0	2	± 0.12	8	± 0.41	12	± 0.49
C14:0	4	± 0.40	12	± 0.65	20	± 1.12
C16:0	75	± 2.05	155	± 5.45	260	± 5.02
C16:1	27	± 1.25	20	± 1.41	30	± 1.10
C18:0	3	± 0.12	3	± 0.11	6	± 0.41
C18:1 (vaccenate)	57	± 1.25	83	± 1.48	135	± 2.96
C17:0c	1	± 0.00	1	± 0.00	1	± 0.00
C19:0c	3	± 0.22	36	± 1.87	69	± 2.50
total	172	± 5	318	± 11	533	± 14

Table S2: *Fatty acid distribution in µg/g DCW of Escherichia coli BL21 (DE3) expressing pET28aKASI at different time intervals. T=0: directly before induction with IPTG, t=12: 12 hours after induction with IPTG, t=24: 24 hours after induction with IPTG. All fatty acid values are the average of at least three biological replicates with the associated standard deviation indicated.*

BL21 (DE3) pET28a KASI						
Fatty acid distribution (µg/g)	T=0		T=12		T=24	
C12:0	4	± 0.12	25	± 1.16	20	± 0.67
C14:0	5	± 0.05	24	± 1.06	18	± 0.59
C16:0	65	± 4.40	291	±12.76	483	± 16.8
C16:1	27	± 1.27	80	± 3.37	132	± 5.80
C18:0	3	± 0.07	17	± 0.52	27	± 1.42
C18:1 (vaccenate)	47	± 1.95	228	± 7.29	349	± 8.62
C17:0c	1	± 0.00	2	± 0.10	5	± 0.88
C19:0c	3	± 0.09	21	± 0.30	34	± 1.20
Total	155	± 8	688	± 27	1068	± 36

Table S3: *Fatty acid distribution in μg/g DCW of Escherichia coli BL21 (DE3) ΔfadD pET28a- versus BL21 (DE3) ΔfadD expressing pET28aKASI and pET28aKASII separately. Samples were collected 48 hours after induction with IPTG. All fatty acid values are the average of at least three biological replicates with the associated standard deviation indicated.*

**BL21 (DE3)**						
Fatty acid distribution (μg/g)	*ΔfadD-*		*ΔfadD-KASI*		*ΔfadD-KASII*	
C12:0	20	± 0.7	38	± 1.6	20	± 0.6
C14:0	44	± 1.2	66	± 2.3	42	± 1.3
C16:0	198	± 9.9	371	± 16.9	221	± 14.3
C16:1	5	± 0.4	168	± 5.8	90	± 3.8
C18:0	3	± 0.2	5	± 0.3	4	± 0.2
C18:1 (vaccenate)	20	± 1.1	188	± 4.7	88	± 3.6
C17:0c	122	± 4.0	63	± 2.7	20	± 0.8
C19:0c	60	± 2.4	13	± 0.4	67	± 1.2
total	471	± 20	912	± 34	557	± 26

Table S4: *Fatty acid distribution in μg/g DCW of Escherichia coli BL21 (DE3) ΔfadF pET28a- versus BL21 (DE3) ΔfadD expressing pET28aKASII. Samples were collected 48 hours after induction with IPTG. All fatty acid values are the average of at least three biological replicates with the associated standard deviation indicated.*

**BL21DE3**				
Fatty acid distribution (μg/g)	*ΔfabF*		*ΔfabF- KASII*	
C12:0	13	± 0.3	12	± 0.3
C14:0	34	± 0.4	28	± 0.8
C16:0	164	± 1.2	148	± 4.3
C16:1	40	± 0.4	114	± 3.1
C18:0	3	± 0.1	7	± 0.2
C18:1 (vaccenate)	7	± 0.0	27	± 0.8
C17:0c	137	± 0.7	35	± 1.2
C19:0c	0	± 0.0	0	± 0.0
Total	397	± 2.8	370	± 10.7

Table S5: *Fatty acid distribution in µg/mg DCW of the 1.3 Liter fermentation of Escherichia coli BL21 (DE3) ΔfadD expressing an empty pET28a vector, pET28aKASI and pET28aKASII respectively. Samples were collected 48 hours after induction with IPTG. All fatty acid values are the average of at least three biological replicates with the associated standard deviation indicated.*

**BL21DE3**						
**Fatty acid distribution (µg/mg)**	*ΔfadD*		*ΔfadD-KASI*		*ΔfadD-KASII*	
C12:0	7	± 0.2	12	± 0.2	9	± 0.1
C14:0	16	± 0.7	26	± 0.8	20	± 0.2
C16:0	78	± 2.7	137	± 2.9	104	± 1.1
C16:1	6	± 0.3	23	± 0.4	8	± 0.1
C18:0	6	± 0.1	18	± 0.1	9	± 0.1
C18:1 (vaccenate)	16	± 0.5	41	± 0.8	29	± 0.3
C17:0c	7	± 0.1	9	± 0.2	11	± 0.1
C19:0c	20	± 0.4	26	± 0.3	31	± 0.4
total	157	± 5	292	± 6	222	± 2

Table S6: *Fatty acid distribution in µg/mg DCW of the 1.3 Liter fermentation of Escherichia coli BL21 (DE3) ΔfabF expressing pET28aKASI and pET28aKASII respectively. Samples were collected 48 hours after induction with IPTG. All fatty acid values are the average of at least three biological replicates with the associated standard deviation indicated.*

**BL21DE3**				
**Fatty acid distribution (µg/mg)**	*ΔfabF-KASI*		*ΔfabF-KASII*	
C12:0	10	± 0.28	4	± 0.14
C14:0	20	± 0.51	15	± 0.29
C16:0	97	± 0.81	105	± 0.47
C16:1	17	± 0.12	12	± 0.09
C18:0	12	± 0.04	5	± 0.05
C18:1 (vaccenate)	3	± 0.06	9	± 0.18
C17:0c	7	± 0.42	5	± 0.41
C19:0c	2	± 0.04	6	± 0.09
total	167	± 2.28	162	± 1.71

Table S7: *Growth analysis of M9 minimal media shake flask studies of wild type Escherichia coli BL21 (DE3) expressing pET28a-, pET28aKASI and pET28aKASII respectively. Absorbance values (OD600) are the average of at least three biological replicates.*

M9 media	BL21 (DE3) pET28a-			BL21 (DE3) pET28a-KASI			BL21 (DE3) pET28a-KASII		
Time in hours	Absorbance	Exponential growth	Specific growth rate	Absorbance	Exponential growth	Specific growth rate	Absorbance	Exponential growth	Specific Growth rate
0	0.186	-0.73048706	0.32850407	0.162	-0.79048499	0.42985656	0.178	-0.74958	0.29328433
1	0.25833333	-0.58781955	0.58921933	0.249	-0.60380065	0.62887636	0.23866667	-0.62220823	0.61774916
2	0.46566667	-0.33192485	0.685242	0.467	-0.33068312	0.68454498	0.44266667	-0.35392318	0.67337467
3	0.924	-0.03432803	0.44229647	0.926	-0.03338901	0.44568217	0.868	-0.06148027	0.48515327
4	1.438	0.15775889	0.21896236	1.446	0.16016829	0.23003538	1.41	0.14921911	0.23862592
5	1.79	0.25285303	0.25719407	1.82	0.26007139	0.23189634	1.79	0.25285303	0.20624174
6	2.315	0.364551	0.10643985	2.295	0.36078269	0.10536052	2.2	0.34242268	0.12783337
7	2.575	0.41077723	-0.00094884	2.55	0.40654018	-0.0021777	2.5	0.39794001	-0.00172118
23.5	2.535	0.40397796	-0.03410561	2.46	0.39093511	-0.04447024	2.43	0.38560627	-0.05715841
24.5	2.45	0.38916608	-0.00136193	2.353	0.37162193	-0.00085052	2.295	0.36078269	-0.0043716

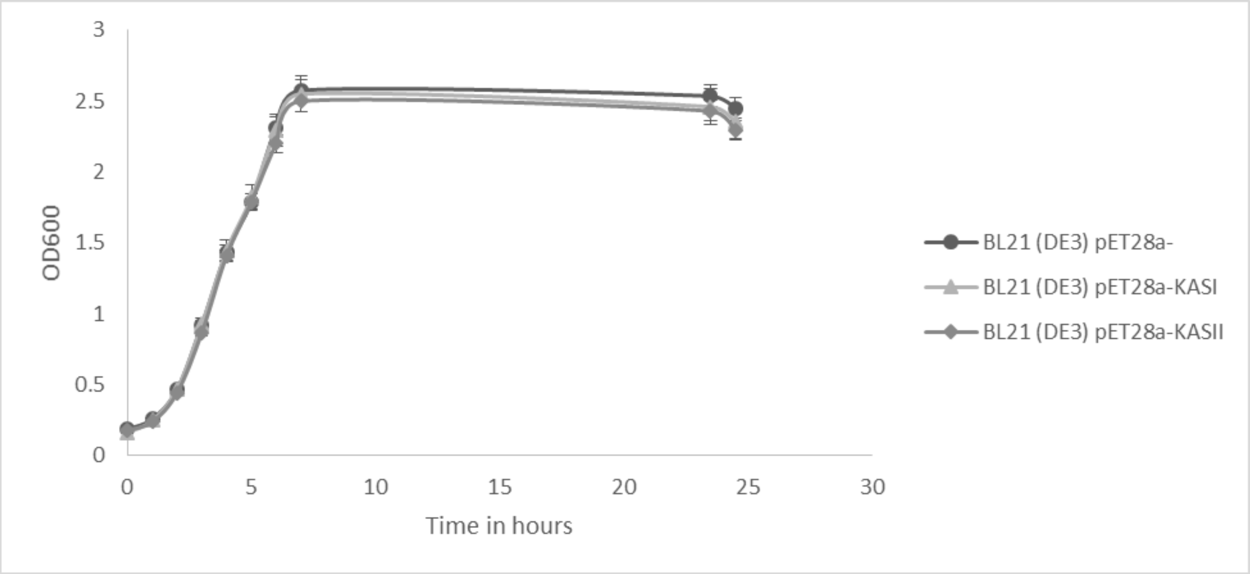

Figure S1: *Growth curve of M9 minimal media shake flask studies of wild type Escherichia coli BL21 (DE3) expressing pET28a-, pET28aKASI and pET28aKASII respectively. Absorbance values (OD600) are the average of at least three biological replicates with the associated standard deviation indicated.*
